# Major Amputation Profoundly Increases Mortality in Patients With Diabetic Foot Infection

**DOI:** 10.3389/fsurg.2021.655902

**Published:** 2021-04-30

**Authors:** Miska Vuorlaakso, Juha Kiiski, Tapani Salonen, Matti Karppelin, Mika Helminen, Ilkka Kaartinen

**Affiliations:** ^1^Faculty of Medicine and Life Sciences, Tampere University, Tampere, Finland; ^2^Department of Surgery, Kanta-Häme Central Hospital, Hämeenlinna, Finland; ^3^Department of Musculoskeletal Surgery and Diseases, Tampere University Hospital, Tampere, Finland; ^4^Department of Internal Medicine, Tampere University Hospital, Tampere, Finland; ^5^Department of Infectious Diseases, Tampere University Hospital, Tampere, Finland; ^6^Tays Research Services, Tampere University Hospital, Tampere, Finland; ^7^Faculty of Social Sciences, Health Sciences, Tampere University, Tampere, Finland

**Keywords:** diabetic foot, risk factors, amputation, morbidity, mortality, survival rate

## Abstract

**Introduction:** An acute diabetic foot infection (DFI) is a serious condition and a leading cause of hospitalization and major amputation in patients with diabetes. Aim of this study was to evaluate the long term survival and risk factors for death and amputation after the DFI requiring hospital treatment.

**Materials and Methods:** A retrospective study included all adult patients hospitalized for DFI treatments during 2010–2014. Overall survival (OS) and amputation free survival (AFS) (without major amputation) was calculated. We performed a Cox regression analysis of several clinical parameters to evaluate the effects of clinical parameters on overall and amputation-free survival.

**Results:** Total of 324 patients with mean age of 66.8 (SD 12.8) years were included. The one- and five-year OS after DFI 81.2% (95%CI 77.5–84.9%) and 49.7% (95%CI 44.8–54.6%), respectively. Major amputation, wound ischemia, older age, and a low glomerular filtration rate reduced the OS after DFI. After a major amputation, the one- and five-year OS was 41.7% (95%CI 13.9–69.5) and 8.3% (95%CI 0.0–24.0%), respectively. Wound ischemia, older age, and elevated C-reactive protein reduced AFS. In contrast, hypertensive medication use was identified as a protective factor.

**Conclusion:** Mortality after a DFI remains high and is significantly increased after a major amputation. Findings highlight the importance of early wound and ischemia management for DFI prevention.

## Introduction

The burden of diabetes and its complications is an increasing problem worldwide. The prevalence of diabetes is likely to grow in coming years, which will lead to increases in deaths and healthcare costs (1). A patient with diabetes is at major risk of developing a foot ulceration, which can lead to infections, extensive hospitalizations, lower-extremity amputations, and even death. It is important to keep up to date on knowledge of factors that can predict mortality and morbidity to meet this global challenge.

The 5-year overall survival of patients with diabetic foot ulcers (DFUs) is 70%, and after a major amputation, it declines to only 43% (2). The most important risk factors for mortality in patients with DFUs are age, ischemia, impaired renal function, and male sex (3–5). Infection is a major complication of DFUs, and long-term survival of these patients remains unknown. Few studies have focused on patients with acute diabetic foot infections (DFIs). A recent study showed that patients with DFIs had 15% mortality at 1 year, and 17% of patients underwent lower extremity amputations (6). In another study, patients with infectious gangrene had 40% survival over 5 years (7). DFIs also have a profoundly negative impact on patient quality of life (8).

A major amputation is a feared consequence of complicated diabetes; it is associated with severe physical impairment (9). Lower extremity amputations, due to diabetic foot complications, were identified as an independent risk factor of premature death (2). Therefore, next to overall survival (OS), amputation-free survival (AFS; that is, survival without amputations above the ankle) is an important outcome for patients with DFI.

The Laboratory Risk Indicator for Necrotizing Fasciitis score (LRINEC score) is a diagnostic scoring system that was developed to distinguish necrotizing fasciitis from other soft tissue infections (10). Originally, the LRINEC cutoff score for identifying necrotizing fasciitis was defined as 6; however, a more recent study indicated that a LRINEC score of 8 or higher was more sensitive for patients with diabetes (11). We investigated whether the LRINEC score could be used as an indicator of severe DFI and a poor prognosis.

The primary aim of this study was to investigate the long-term survival and risk factors for OS and AFS in patients with DFI that required inpatient treatment in a tertiary university hospital.

## Materials and Methods

This retrospective cohort study was conducted at Tampere University Hospital (Institutional Review Board approval ETL-code: R14545S). All adult patients hospitalized with an acute DFI during 2010–2014 were included in the study. This population and patient selection process was described in an earlier study in detail (12). Compared to the previous study cohort, we excluded one patient that was under 18 years of age.

From the hospital records, we collected data on patient demographics (age and gender), registered diagnoses (ischemic heart disease, chronic obstructive pulmonary disease, congestive heart failure and dyslipidemia), microbiological and clinical chemistry findings, surgical revisions and amputations, open and endovascular revascularization procedures, and the length of hospital stay. Wound status was assessed from the patient records, and wounds were staged according to the University of Texas Staging System for Diabetic Foot Ulcers (UT scale) by a specialized plastic surgeon (13). For the analysis, wounds were classified separately, based on wound perfusion (ischemic vs. non-ischemic) and wound depth (1: superficial, 2: penetrating tendon or capsule, 3: penetrating joint or bone). We calculated the LRINEC score from the first laboratory results after admission, and we chose a cutoff score of 8 or higher to identify necrotizing fasciitis. The interval from admission to surgery was defined as the number of days from admission to a first surgical revision. The glomerular filtration rate (GFR) was calculated with the Modification of Diet in Renal Disease equation (14). Hypertension was defined as the use of antihypertensive medication.

The mean and standard deviation (SD) were calculated for normally distributed continuous variables. The median and interquartile range (IQR) were calculated for continuous variables with skewed distributions. We performed a Cox regression analysis to evaluate the significance of associations of different variables with OS and AFS. In the AFS analysis, the endpoint was defined as death or a major amputation (above the ankle), and in the OS analysis, the endpoint was death. AFS analysis was limited to 2 years following hospitalization, since most patients had censored out after this time. Time of follow up was considered to end in last visit to hospital. However, OS was reliably analyzed during 5 years, since the data of death dates was obtained from national registry. All variables were first analyzed individually with Cox regression. For the Cox regression, we included the GFR, hemoglobin A1c (HbA1c), C-reactive protein (CRP), and leukocyte levels as continuous variables without thresholds. Then, all variables that were individually significantly associated with survival were included into a multivariable Cox regression analysis. For variables that showed a significant association with survival in the multivariable analysis, we performed Kaplan-Meier analyses and reviewed survival tables. For these analyses, age and GFR were divided into two groups. For age, the median (67.0 years) was used as a cutoff value. For GFR, a value of 60.0 ml/min was chosen as a cutoff value, because this value served as the threshold between mild and moderate kidney disease; in addition, it was close to the median (58.6 ml/min). Statistical analyses were planned and reviewed with a professional statistician. All analyses were performed with SPSS software (IBM SPSS Statistics, Version 24.0. Armonk, NY).

## Results

We enrolled 324 patients (237 males, 87 females; mean age, 66.8 years, SD 12.8) with 404 periods of hospitalization. Their comorbidities, wound characteristics, and bacterial findings are presented in Table 1. A case was defined as a period of hospitalization.

**Table 1:** Comorbidities, wound characteristics, and bacterial culture findings.

**Comorbidities**	***n* (%)**
Hypertension	188 (46.5)
Ischemic heart disease	113 (28)
Impaired kidney function (GFR <60 ml/min)	157 (38.9)
Chronic obstructive pulmonary disease	21 (5.2)
Congestive heart failure	84 (20.8)
Dyslipidemia	25 (6.2)
Ulcer type and depth^*^	
non-ischemic wound infection (B)	164 (40.6)
ischemic wound infection (D)	239 (59.2)
ulcer type undefined	1 (0.2)
superficial ulcer (1)	83 (20.5)
penetrating to tendon or capsule (2)	61 (15.1)
penetrating to joint or bone (3)	258 (63.9)
ulcer depth undefined	2 (0.5)
**Bacterial cultures in wound**	
*Staphylococcus aureus*	77 (19.1)
MRSA	10 (2.5)
Gram-negative rods	60 (14.9)
Beta hemolytic streptococci	51 (12.6)
Coagulase-negative staphylococci	20 (5)

* *University of Texas Wound Classification System of Diabetic Foot Ulcers; GFR, glomerular filtration rate; (B), (D), ulcer type classifications; (1), (2), (3), ulcer depth classifications; MRSA, methicillin-resistant Staphylococcus aureus*.

The median hospitalization time was 6 days (IQR: 4–10). Surgical debridement (wound debridement or amputation) was performed in 208 cases. The median interval from admission to the first surgical intervention was 2 days (IQR: 1–4). Revascularization within 1 month of admission, was performed in 65 cases. Open revascularization procedure was performed in 17 cases, endovascular revascularization in 45 cases, and both in 3 cases. Median interval from admission to revascularization was 3 days (IQR: 1.5–6). Table 2 summarizes the distribution of laboratory test results among the cases. Data for computing the LRINEC score were available in 305 cases. The LRINEC scores (mean: 4.6, SD 3.0) were over 6 in 118 (38.7%) cases, and over 8 in 62 (20.3%) cases.

**Table 2:** Distribution of laboratory test results among the cases.

	**Cases**	**Median**	**IQR**
GFR (ml/min)	304	58.6	36.1–88.7
HbA1c (mmol/mol)	262	62	51–74
CRP (first week highest, mg/l)	310	150.8	87.3–231.5
Leukocytes (first week highest, E9/l)	316	13.4	10.1–17

**Figure 1:**
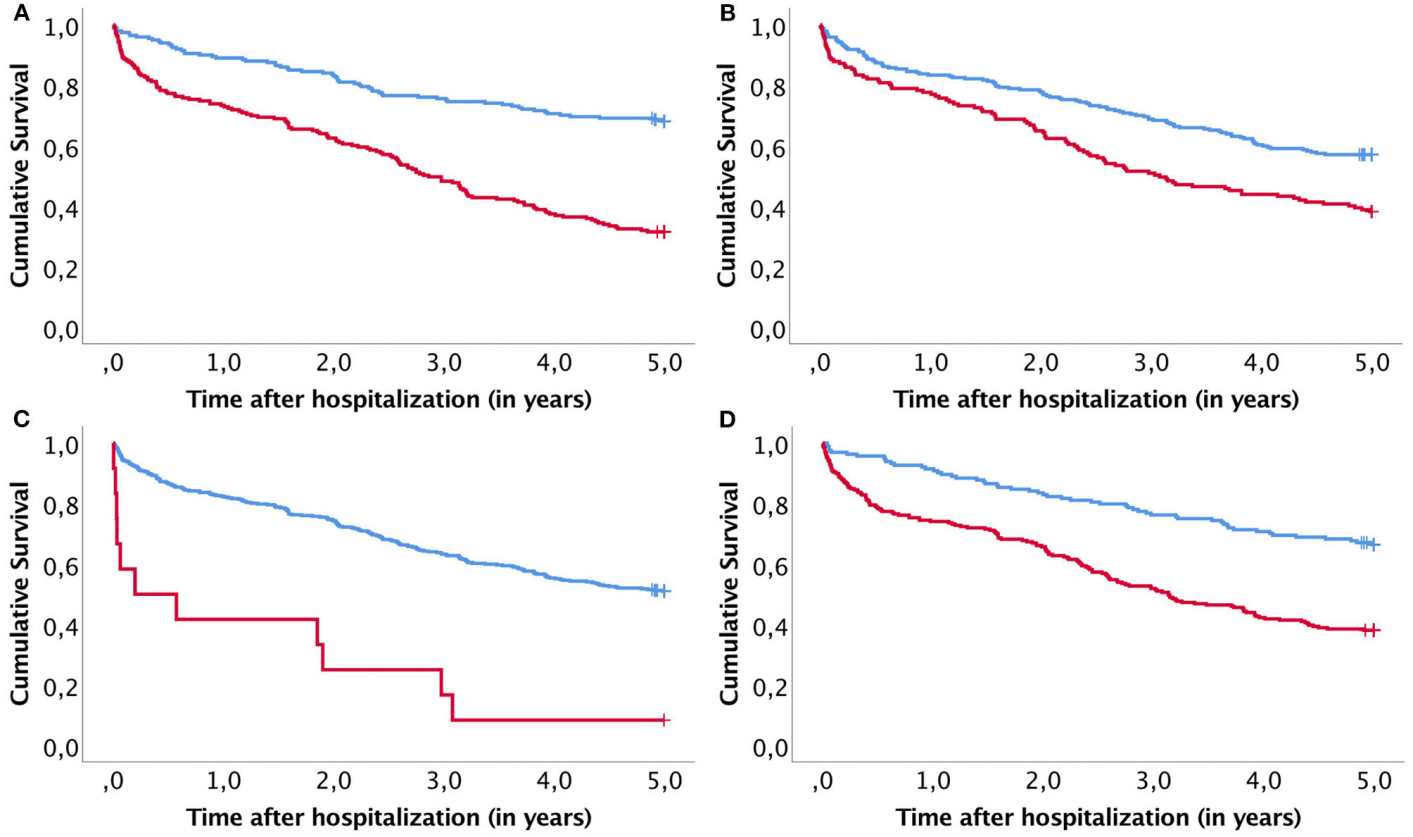
Kaplan–Meier curves compare survival between subgroups identified with a significant risk factor. **(A)** Age under (blue line) or over (red line) 67 years; **(B)** GFR under (red line) or over (blue line) 60 ml/min; **(C)** individuals with (red line) or without (blue line) a major amputation; and **(D)** individuals with (red line) or without (blue line) wound ischemia.

The OS rates for the entire population after one, three, and 5 years were 81.2% (95%CI: 77.5–84.9%), 61.9% (95%CI: 57.2–66.6%), and 49.7% (95%CI: 44.8–54.6%), respectively. Among patients that underwent a major amputation, these survival rates were 41.7% (95%CI: 13.9–69.5%), 16.7% (95%CI: 0.0–37.9%), and 8.3% (95%CI 0.0–24.0%), respectively. In the Cox regression analysis, we found that four factors were associated with a significantly shorter OS: a major amputation during hospitalization, ischemia with an infected ulcer, age over 67 years, and a GFR under 60 ml/min (Figure 1).

The univariate Cox regression analysis identified six factors that significantly reduced the OS: a major amputation, wound ischemia, revascularization procedure within a month of admission, ischemic heart disease, congestive heart failure and age. In contrast, male gender, a high GFR, and a low HbA1c increased the OS (Supplementary Table 1). The multivariable model showed that a major amputation, wound ischemia, age, and GFR remained significant factors (Table 3). A univariate analysis showed that AFS was reduced in patients with wound ischemia, deep wounds (to the bone or joint), age, high leukocyte counts, and high CRP levels. In contrast, AFS was increased in patients that used hypertension medications (Supplementary Table 1). The multivariable analysis showed that wound ischemia, age, CRP, and hypertension medication use retained significance (Table 4). Neither the OS nor the AFS was significantly affected by bacterial culture findings, interval from admission to surgery, or the LRINEC-score.

**Table 3:** Multivariable Cox regression analysis of overall survival (*N* = 247).

**Influencing factor**	***P***	**HR**	**95% CI**
Male (vs. Female)	0.470	1.173	0.761–1.807
Age	** <0.001**	**1.055**	**1.035**–**1.076**
Wound ischemia (vs. non-ischemic wound)	**0.046**	**1.598**	**1.008**–**2.532**
Major amputation	** <0.001**	**6.673**	**2.836**–**15.700**
Revascularization (within 1 month)	0.720	1.091	0.678–1.755
Ischemic heart disease	0.208	1.321	0.856–2.038
GFR (ml/min)	** <0.001**	**0.989**	**0.982**–**0.995**
HbA1c (mmol/mol)	0.832	0.999	0.987–1.011
Congestive heart failure	0.237	1.310	0.837–2.049

*GFR, glomerular filtration rate; HbA1c, glycated hemoglobin*.

*Variables significant in multivariable model are bolded*.

**Table 4:** Multivariable Cox regression analysis of amputation-free survival (*N* = 308).

**Influencing factor**	***P***	**HR**	**95% CI**
Age	**0.001**	**1.033**	**1.014**–**1.052**
Wound ischemia (vs. non-ischemic wound)	** <0.001**	**3.922**	**2.219**–**6.933**
Ulcer penetrating to joint or bone	0.077	1.742	0.943–3.221
Hypertension	**0.004**	**0.533**	**0.349**–**0.814**
CRP (first week highest, mg/l)	**0.007**	**1.003**	**1.001**–**1.005**
Leukocytes (first week highest, E9/l)	0.073	1.035	0.997–1.074

*CRP, C-reactive protein*.

*Values significant in multivariable model are bolded*.

**Figure 2:**
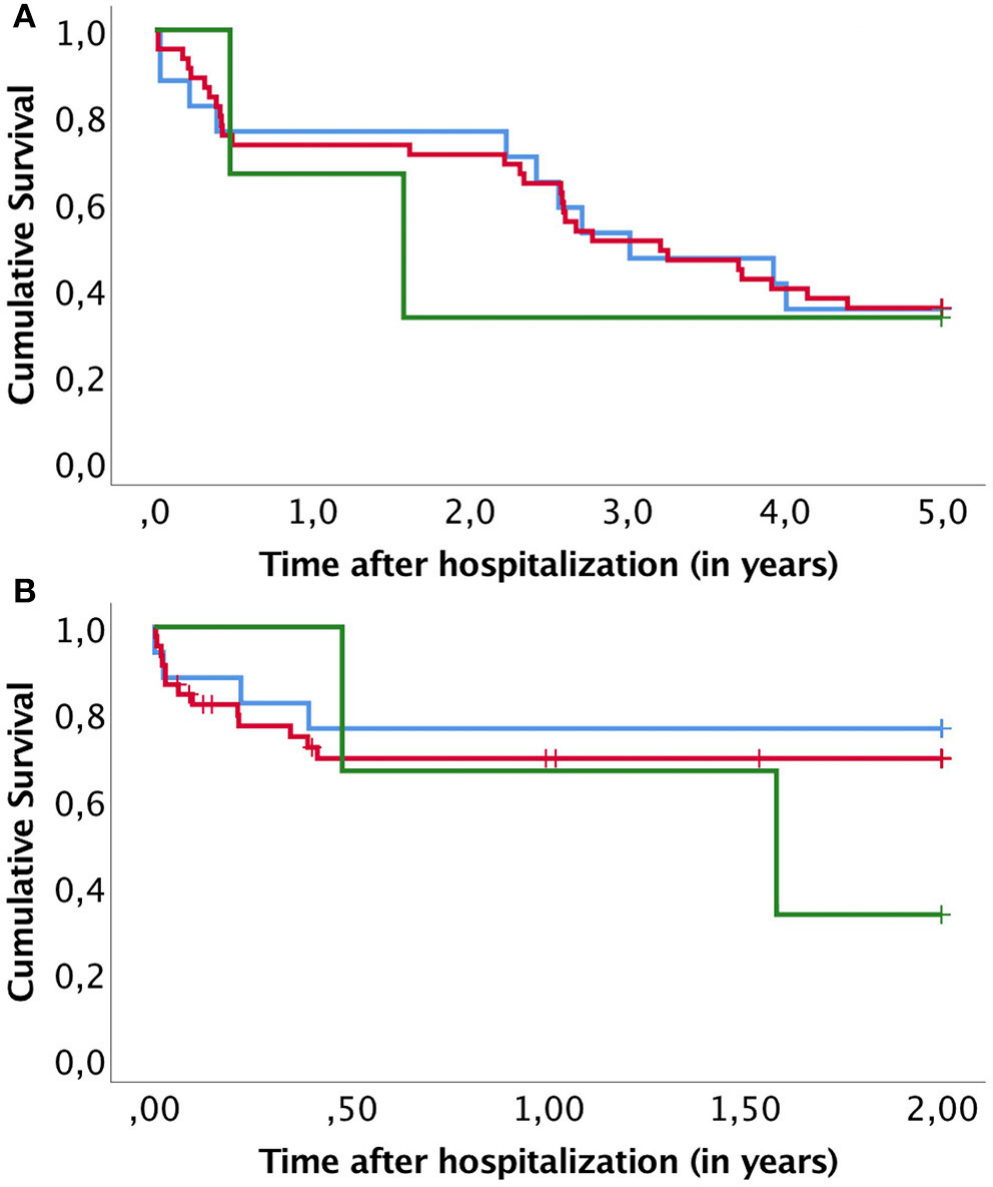
Kaplan–Meier curves compare overall survival **(A)** and amputation free survival **(B)** after different revascularization approaches. Groups presented are open revascularization procedure (blue line), endovascular revascularization (red line) and both open and endovascular procedure (green line).

**Figure 3:**
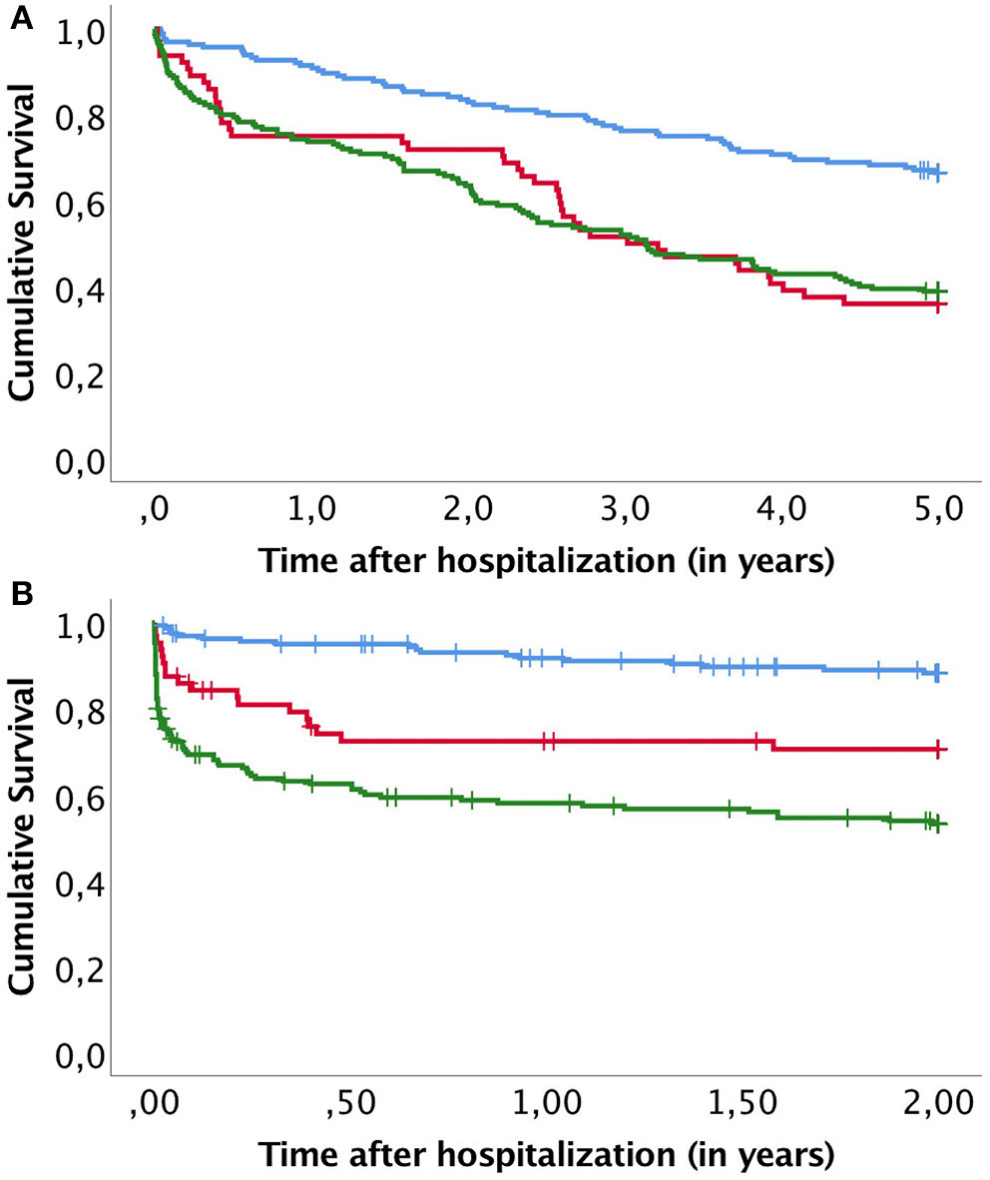
Kaplan–Meier curves compare overall survival **(A)** and amputation free survival **(B)** between non-ischemic infection (blue line), ischemic infection receiving revascularization (red line) and ischemic infection and no revascularization (green line).

A Kaplan-Meier subanalysis was performed to further evaluate the role of revascularization on survival. OS and AFS were compared within groups receiving open, endovascular or both revascularizations (Figure 2). There was no significant difference in OS (*p* = 0.956) nor AFS (*p* = 0.554). In addition, a subanalysis to compare survival between non-ischemic infection and ischemic infection with and without revascularization was conducted (Figure 3). OS was similar after ischemic infection with and without revascularization. However, AFS was improved after revascularization compared to ischemic infection without revascularization. This difference remained statistically significant, when a Kaplan–Meier analysis was performed only to ischemic infection as a subgroup.

## Discussion

This study showed that survival after a DFI was poor, even in a tertiary university hospital setting with advanced treatment resources. Age, wound ischemia, major amputations, and impaired kidney function were the most important predictors of a reduced OS. Moreover, age, wound ischemia, and high CRP were associated with a reduced AFS; however, use of a hypertensive medication seemed to increase the AFS.

The one-year OS after hospitalization due to a DFI was 81.2%. This result was similar to that found in a recent prospective multi-center study in England, where 15% mortality was observed during the first year after a DFI (6). Diabetes is estimated to be related to 31.4% of deaths in Europe (1). In our study, half of the patients died within 5 years after a DFI. This finding highlighted the serious nature of a DFI. For comparison, the 5-year mortality after a DFI was larger than the mortality after an ST-elevation myocardial infarction without any reperfusion (41%) and similar to mortality after a stroke (48%) or among patients undergoing hemodialysis (51%) (15–17).

Among patients that underwent a major amputation, the risk of death after the infection was nearly six-fold that of patients with infections that could be managed without amputation. After a 5-year follow-up, only 1 of 12 patients (8.3%) survived. We found that a lower OS was partially associated with age and comorbidities, such as foot ischemia and impaired renal function. However, a major amputation remained a significant factor, when the multivariable analysis was adjusted for these comorbidities; thus, a major amputation should be considered an independent risk factor for death. A previous study of patients with DFIs found that mortality was associated with minor and major amputations and ischemia (2). In contrast, our data did not show an increased risk of death after a minor amputation. We interpreted this result as an indication that, in the face of an acute infection, a minor amputation could control the infection, avoid a major amputation, and save the limb.

The AFS was greatly reduced in patients with ischemic wounds. Ischemia and infections are considered the two most threatening factors for a diabetic foot prognosis (18). Revascularization is the cornerstone of treatment, but patients with an ischemic DFI should be given a comprehensive treatment plan for treating both the infection and other comorbidities (19). In our study, 16.1% of the cases had revascularization within 1 month of admission and in most cases within 1 week. We found no difference in survival between patients receiving open or endovascular revascularization (Figure 2). In addition, revascularization did not affect OS after the infection. However, AFS within patients with ischemic infection was significantly improved by revascularization (Figure 3). This shows that timely management of ischemic in DFI is crucial to improve limb salvage among patients surviving DFI. Therefore, early involvement of vascular surgery in treatment of acute diabetic foot is strongly indicated.

In our cohort, a higher CRP (indicating a more severe infection) was also associated with a worse AFS. In the univariate analysis, we found that the AFS was also associated with a deep wound (penetrating to the bone or joint) and high blood leukocytes. However, these factors lost significance in the multivariable analysis. A deep wound often develops over a long time, and it can go unnoticed with lack of proper foot care and diabetic neuropathy. Long-lasting and multiple foot ulcers were associated with a worse outcome (6). This observation highlighted the importance of detecting the infection early and providing sufficient interventions in treating diabetic foot ulcers.

Our results showed that use of a hypertensive medication was associated with an increased AFS. Hypertension has been identified as a risk factor for major amputations among patients with diabetes (20). However, previous studies have shown that hypertension did not impact major amputation rates (21). In our study, we lacked data on blood pressure; therefore, we could only analyze the association between the use of hypertensive medication and AFS. Although our finding is interesting, it requires more targeted research for adequate interpretation.

A major amputation due to a DFI causes serious physical impairment (9). Therefore, a reasonable goal is to improve the survival of patients with diabetes without performing a major amputation. In some cases, a major amputation is the best option, and it can improve the quality of life (22). This quandary emphasizes the importance of a careful preoperative assessment for determining whether limb salvage or a major amputation would be more beneficial to the patient.

The major strengths of this study were the inclusion of all hospitalized DFI cases in our area and the long-term follow-up. The retrospective nature of the study was an inevitable limitation. Our data did not include features as ankle-brachial index or clinical severity of infection, which are included in most recent scales. Nevertheless, we could classify all but two wounds retrospectively using the UT scale. Other study limitations included the fact that complete data on covariates were not available for all cases and there may be some inconsistency in registered diagnosis of co-morbidities. Moreover, the population of this study comprised only hospitalized patients in a tertiary care hospital, although DFIs are also treated in primary and secondary hospitals and in outpatient care. On the other hand, this might also be viewed as a study strength, because we focused on the most severe DFIs.

In conclusion, patients with DFIs comprise a group associated with high mortality and morbidity. A major amputation often precedes death; therefore, treatments should aim to improve both the AFS and OS. Ischemic infections in deep wounds, combined with a high CRP level is the worst case scenario for a DFI; hence preventing this scenario merits robust action.

## Data Availability Statement

The raw data supporting the conclusions of this article will be made available by the authors, without undue reservation.

## Author Contributions

MV: conceptualization, methodology, software, formal analysis, investigation, writing original draft. JK: conceptualization, writing review and editing, visualization, supervision. TS and MK: writing review and editing. MH: methodology, software, writing review and editing, visualization. IK: conceptualization, resources, writing review and editing, supervision, funding acquisition. All authors contributed to the article and approved the submitted version.

## Conflict of Interest

The authors declare that the research was conducted in the absence of any commercial or financial relationships that could be construed as a potential conflict of interest.
