# Supplementary material for: Major Amputation Profoundly Increases Mortality in Patients With Diabetic Foot Infection
Source: Front Surg. 2021 Apr 30;8:655902. doi: 10.3389/fsurg.2021.655902 (PMC8120024; doi:10.3389/fsurg.2021.655902)
Supplement: Supplementary file 1 [file Table_1.DOCX]

**Table S1:**

|  |  |  | Overall survival (OS) | | |  | Amputation free survival (AFS) | | |
| --- | --- | --- | --- | --- | --- | --- | --- | --- | --- |
| Factor | n* |  | p** | HR | 95% CI |  | p** | HR | 95% CI |
| Male (vs. Female) | 404 |  | **0.015** | **0.69** | **0.511-0.930** |  | 0.469 | 0.859 | 0.568-1.297 |
| Age | 404 |  | **<0.001** | **1.054** | **1.042-1,067** |  | **<0.001** | **1.038** | **1.022-1.054** |
| Wound ischemia (vs. non-ischemic wound) | 403 |  | **<0.001** | **2.417** | **1.772-3.296** |  | **<0.001** | **4.724** | **2.853-7.822** |
| Wound depth | 402 |  |  |  |  |  |  |  |  |
| 1 |  |  | 0.016 | 1 |  |  | 0.001 | 1 |  |
| 2 |  |  | 0.454 | 0.82 | 0.487-1.380 |  | 0.72 | 0.859 | 0.376-1.964 |
| 3 |  |  | 0.057 | 1.421 | 0.989-2.040 |  | **0.003** | **2.276** | **1.317-3.933** |
| Minor amputation | 404 |  | 0.395 | 0.801 | 0.481-1.335 |  | 0.354 | 0.712 | 0.347-1.460 |
| Major amputation | 404 |  | **<0.001** | **4.373** | **2.373-8.058** |  | - | - | - |
| Revascularization (within one month) | 404 |  | **0.012** | **1.543** | **1.098-2.168** |  | 0.882 | 1.038 | 0.634-1.699 |
| Ischemic heart disease | 404 |  | **<0.001** | **1.927** | **1.447-2.567** |  | 0.554 | 1.128 | 0.757-1.679 |
| Chronic obstructive pulmonary disease | 404 |  | 0.092 | 0.627 | 0.364-1.080 |  | 0.871 | 1.071 | 0.471-2.436 |
| Congestive heart failure | 404 |  | **<0.001** | **2.125** | **1.576-2.866** |  | 0.196 | 0.723 | 0.442-1.183 |
| Dyslipidemia | 404 |  | 0.149 | 0.612 | 0.313-1.193 |  | 0.477 | 0.742 | 0.326-1.689 |
| GFR (ml/min) | 304 |  | **<0.001** | **0.989** | **0.984-0.994** |  | 0.53 | 0.998 | 0.992-1.004 |
| Hypertension | 404 |  | 0.594 | 1.078 | 0.818-1.419 |  | **0.037** | **0.668** | **0.458-0.976** |
| LRINEC ≥ 8 | 305 |  | 0.193 | 0.756 | 0.496-1.152 |  | 0.472 | 1.19 | 0.741-1.913 |
| Delay to first surgery | 208 |  | 0.161 | 0.961 | 0.909-1.016 |  | 0.154 | 0.948 | 0.882-1.020 |
| SA in wound | 404 |  | 0.9 | 0.977 | 0.684-1.396 |  | 0.757 | 0.927 | 0.572-1.501 |
| GNR in wound | 404 |  | 0.93 | 1.017 | 0.694-1.491 |  | 0.349 | 1.259 | 0.777-2.040 |
| MRSA in wound | 404 |  | 0.965 | 1.02 | 0.420-2.478 |  | 0.366 | 1.584 | 0.584-4.296 |
| BHS in wound | 404 |  | 0.095 | 0.675 | 0.425-1.070 |  | 0.082 | 0.546 | 0.277-1.079 |
| CONS in wound | 404 |  | 0.328 | 0.703 | 0.347-1.425 |  | 0.168 | 0.446 | 0.142-1.404 |
| CONS in bone | 404 |  | 0.225 | 0.493 | 0.158-1.543 |  | 0.227 | 0.298 | 0.042-2.131 |
| Bacteria in bone | 404 |  | 0.196 | 0.707 | 0.418-1.196 |  | 0.183 | 0.595 | 0.277-1.278 |
| HbA1c (mmol/mol) | 262 |  | **0.042** | **0.99** | **0.981-1.000** |  | 0.41 | 0.995 | 0.984-1.007 |
| CRP (first week highest, mg/l) | 310 |  | 0.893 | 1 | 0.999-0.002 |  | **0.032** | **1.002** | **1.000-1.004** |
| Leukocytes (first week highest, E9/l) | 316 |  | 0.092 | 1.023 | 0.996-1.050 |  | **0.002** | **1.046** | **1.016-1.076** |

* number of cases in which data was available

** Cox regression analysis

SA, Staphylococcus aureus; GNR, gramnegative rods; MRSA, methicillin-resistant staphylococcus aureus; BHS, beta-hemolytic streptococci; CONS, coagulase negative staphylococci
